# Supplementary material for: Influence of chemical interactions on the electronic properties of BiOI/organic semiconductor heterojunctions for application in solution-processed electronics
Source: J Mater Chem C Mater. 2023 Dec 21;12(4):1366–76. doi: 10.1039/d3tc03443g (PMC10809049; doi:10.1039/d3tc03443g)
Supplement: TC-012-D3TC03443G-s001 [file TC-012-D3TC03443G-s001.pdf]

Electronic Supplementary Material (ESI) for Journal of Materials Chemistry C.
This journal is © The Royal Society of Chemistry 2023

Influence of chemical interactions on the electronic properties of BiOI/organic semiconductor heterojunctions for application in solution-processed electronics

Vaidehi Lapalikar, Preetam Dacha, Mike Hambsch, Yvonne J. Hofstetter, Yana Vaynzof, Stefan C. B. Mannsfeld, Michael Ruck**

** Corresponding authors*

Contents

Experimental Methods ... 2
BiOI thin film fabrication ... 2
OSC/BiOI heterojunction fabrication ... 2
Characterization ... 3
1. PXRD ... 3
2. SEM, EDX and AFM ... 3
3. XPS and UPS ... 3
4. UV-Vis-NIR spectroscopy ... 4
5. GIWAXS ... 4
6. Contact angle measurement ... 4
7. FET measurements ... 5
OSC / BiOI bilayers ... 6
SEM ... 6
AFM ... 7
UV-vis-NIR spectroscopy ... 8
Device measurements ... 8

Experimental Methods

BiOI thin film fabrication

A 0.2 mol L^{-1} solution of BiI_3 in THF was prepared by stirring 117.9 mg of BiI_3 (99.9 % Sigma Aldrich, sublimated at 300 °C) in 1 ml THF (≥ 99,7% HPLC grade, VWR Chemicals) at RT for 2 hours. The solution was then filtered through a 0.2 µm PTFE syringe-filter before being used for coating. The required substrates for characterizations or devices were cleaned thoroughly before use by ultrasonicating them successively in distilled water, acetone and isopropanol for 10 minutes each followed by drying under an N_2 stream. The substrates were then activated in a UV-ozone chamber for 10 minutes. BiI_3 solution was dropped in variable amounts depending on the size of the substrates to ensure complete coverage and spin coated (Polos SPS150i spin coater) for 20 s at 2000 rpm and maximum acceleration. The resulting film was dried at 120 °C for 30 s on a hotplate to remove residual solvent. The conversion of BiI_3 to BiOI was achieved by immersing the BiI_3-coated substrate for 3 minutes in a 1 : 1 vol. H_2O : MeOH bath having a total volume of 40 mL. A visual change can be noted in the film as the color changes from metallic dark gray (BiI_3) to orange (BiOI). The film was dried using N_2, followed by washing with MeOH to remove traces of BiI_3 from it. Lastly, the film was annealed on a hotplate in air at 120 °C for 10 minutes.

OSC/BiOI heterojunction fabrication

The OSC layer was coated from solutions in chloroform with concentrations as 10 mg mL^{-1} for DPPDTT and PDPP4T, 8 mg mL^{-1} for PCDTPT and 5 mg mL^{-1} for P3HT and TIPS pentacene using shear coating. The blade angle was set at 8° with a gap of 50 µm between the edge of the blade and the substrate. 7 µL of precursor solution for 1.5 × 0.5 inch substrates was injected between the substrate and the blade. During coating, the substrate was maintained at room temperature (25 °C) and the coating speed was 1000 µm s^{-1} for DPPDTT, PDPP4T and PCDTPT and 500 µm s^{-1} for P3HT and TIPS pentacene which were the in-house optimized conditions to fabricate uniform thin films. After coating, the samples were transferred on to a hot plate set at 150 ℃ for 10 minutes. For heterojunction devices with Si/SiO_2/(Cr)Au layers, heavily n-doped silicon wafers with 300 nm thick SiO_2 layer as dielectric were used as substrates for fabricating transistor devices. Prior to use, the substrates were cleaned thoroughly as mentioned above. BGBC transistor configuration was realized by evaporating 2.5 nm of Cr and 50 nm of Au for the source-drain electrodes by thermal evaporation using shadow masks under ultrahigh vacuum of 10^{-7} mbar with a deposition rate of 1.5 Å s^{-1}. OSC films were shear-coated on these samples using conditions optimized for the respective OSCs to obtain uniform films. BiOI was spin coated on these OSC films as per the coating procedure described above.

Characterization

1. *PXRD*

Powder X-ray diffraction (PXRD) was performed with a PANalytical Empyrean diffractometer equipped with a Johannson Ge (111) monochromator in Bragg–Brentano geometry using Cu-$K\alpha_1$ radiation (λ = 154.0598 pm). The data was recorded for BiOI thin film fabricated on a 1 × 1 cm^2 glass substrate and measured at a step-size of $\Delta(2\theta)$ = 0.013° in the range 5° ≤ 2θ ≤ 90°. A variable divergence slit was used and the irradiated length of 8 mm was chosen to acquire data with accurate intensity at higher angles.

2. *SEM, EDX and AFM*

Scanning electron microscopy (SEM) was performed using an SU8020 electron microscope (Hitachi) with a triple detector system for secondary and low-energy backscattered electrons. Typically, an acceleration voltage (U_a) of 2 kV with a current (I_e) of 5 µA was used to acquire top-view and cross-sectional images. Samples of pristine BiOI film and OSC/BiOI heterojunctions prepared on Si /(Cr)Au substrates were measured under N_2-cooling to reduce charging effects. Samples were not sputtered before measurements. The composition of the BiOI thin film was determined by semi-quantitative energy dispersive X-ray analysis (U_a = 15 kV) using a Silicon Drift Detector (SDD) X-MaxN (Oxford Instruments). The data was processed with the AZtec software package (Oxford Instruments, 2013).

Surface texture and roughness of pristine BiOI, pristine OSC films and heterojunctions was obtained in an area of 5 μm^2 with a Flex Axiom (Nanosurf) atomic force microscope (AFM) in the tapping mode using TAP-190Al-G non-conducting tips (Budget Sensors). The images were analyzed using Gwyddion. The thickness of the same films was also measured using a Bruker Nano Dektat XTL profilometer. Thickness of BiOI film formed on various OSCs was calculated by subtracting the pristine OSC film thickness from the thickness of corresponding heterojunction.

3. *XPS and UPS*

Samples were transferred to an ultrahigh vacuum chamber (ESCALAB 250Xi by Thermo Scientific, base pressure: $2 \cdot 10^{-10}$ mbar). XPS measurements were carried out using monochromated Al $K\alpha$ radiation (XR6 source, hν = 1486.6 eV) and a pass energy of 20 eV. XPS was performed on BiOI thin film coated on a Si wafer. Due to the highly textured morphology of the BiOI film, measurements were made on multiple samples. The data presented is an average of 8 measurements. UPS measurements were carried out using a He discharge lamp (hν = 21.2 eV) and a pass energy of 2 eV. UPS was performed for all of the pristine OSCs and BiOI films coated on Si wafers. 3 spots were measured on each sample for consistency and one used for the calculations. The work function (E_ψ) was calculated by subtracting the cutoff values of the secondary photoemission onset (E_{cutoff}) from the photon energy of 21.2 eV, using the formula,

$$E_{\psi} = 21.2 - E_{\mathrm{cutoff}}$$

Valence band maximum (VBM) value with respect to the Fermi level ($E_{VBM,\ onset}$) was extracted from the UPS spectra by extrapolating the slope of the photoemission onset and reading the value from the intersection with the *x*-axis. The VBM value with respect to the vacuum level was then calculated with the formula,

$$E_{VBM} = E_{\psi} + E_{VBM,onset}$$

The measurement error of UPS is approximately 0.1 eV.

4. UV-Vis-NIR spectroscopy

The absorption spectra of pristine BiOI, pristine OSC films and heterojunctions on quartz substrates with the materials being coated as described above were acquired in the wavelength range of 250-3200 nm using the Cary 5000 UV-Vis-NIR spectrophotometer in transmission mode. High quality 2.5 × 2.5 cm^2 quartz substrates (Techinstro Ltd.) were used for the measurements. Samples of pristine BiOI and OSCs along with OSC/BiOI heterojunctions were prepared as per the coating procedures above. Absorbance measurements of pristine samples were further used to calculate the material bandgap using the Tauc relation as follows:

$$(\alpha \mathrm{h\upsilon})^{1/\gamma} = B(\mathrm{h\upsilon} - E_{\mathrm{g}})$$

Where E_{g} is the optical band gap, h Planck's constant, v the frequency of incident photons, B the band tailing parameter, α is the energy-dependent absorption coefficient, and γ is the index, which is 2 for an indirect transition and 1/2 for a direct transition.

5. GIWAXS

Grazing-incidence wide-angle X-ray scattering (GIWAXS) measurements of the BiOI films were performed at BL11 - NCD SWEET at ALBA Synchrotron Facility, Barcelona, Spain. The beam energy was 12.4 keV and the spot size of the beam was 70 µm × 150 µm. A Rayonix LX255HS area detector was placed 167 mm behind the sample. The sample-detector distance and the beam center on the detector were verified using chromium(III) oxide as a reference. The incidence angle of the beam was 0.12° and the sample was exposed for 20 s to the beam. All data were analyzed using WxDiff.

6. Contact angle measurement

The water contact angle of the as-prepared OSC films was measured using an OCA Data Physics contact angle measurement instrument. A constant volume of distilled water was dispensed from the instrument

on to the substrate by regulating the pulse and count values. An average of ~~at least~~ five measurements was taken.

7. FET measurements

Devices were measured with a Keysight B1500 Semiconductor Analyzer in a dark room. The hole field-effect mobility (μ_h) was calculated using the following equation.

$$\mu_h = \frac{2L}{WC_\mathrm{i}}\left(\frac{\partial\sqrt{|I_\mathrm{DS}|}}{\partial V_\mathrm{GS}}\right)^2$$

Here, I_{DS} is the drain current, C_i is the capacitance per unit area of the dielectric, W is the channel width, L is the channel length, and V_{GS} is the gate voltage. The V_{TH} was obtained from the x-intercept of the linear fit of the $\sqrt{|I_\mathrm{DS}|}$ versus V_{GS} curve. In this report, the C_i was 11.5 nFcm^{-2}. The channel width and length for all TFTs was 4500 µm and 200 µm respectively unless otherwise specified.

All fabrication steps and characterizations were performed in ambient air with relative humidity of about 30 % unless otherwise specified.

OSC / BiOI bilayers

SEM

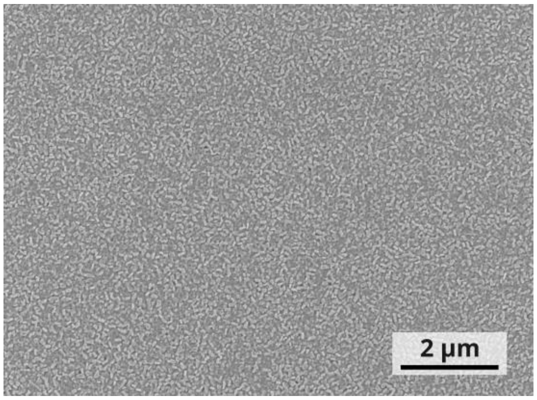

Figure S1. Top view SEM image of the spin coated BiI_3 thin film.

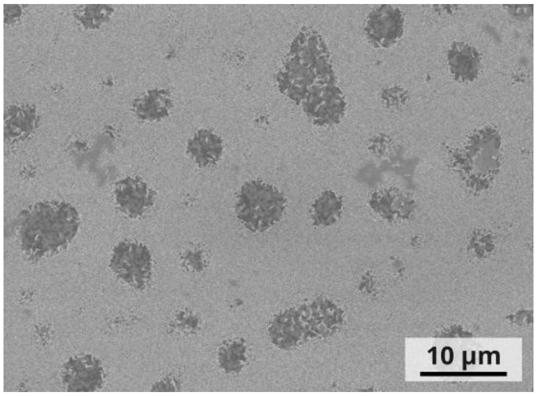

Figure S2. Top view SEM image of PCDTPT/BiOI bilayer showing large areas of horizontally aligned particles throughout the film.

AFM

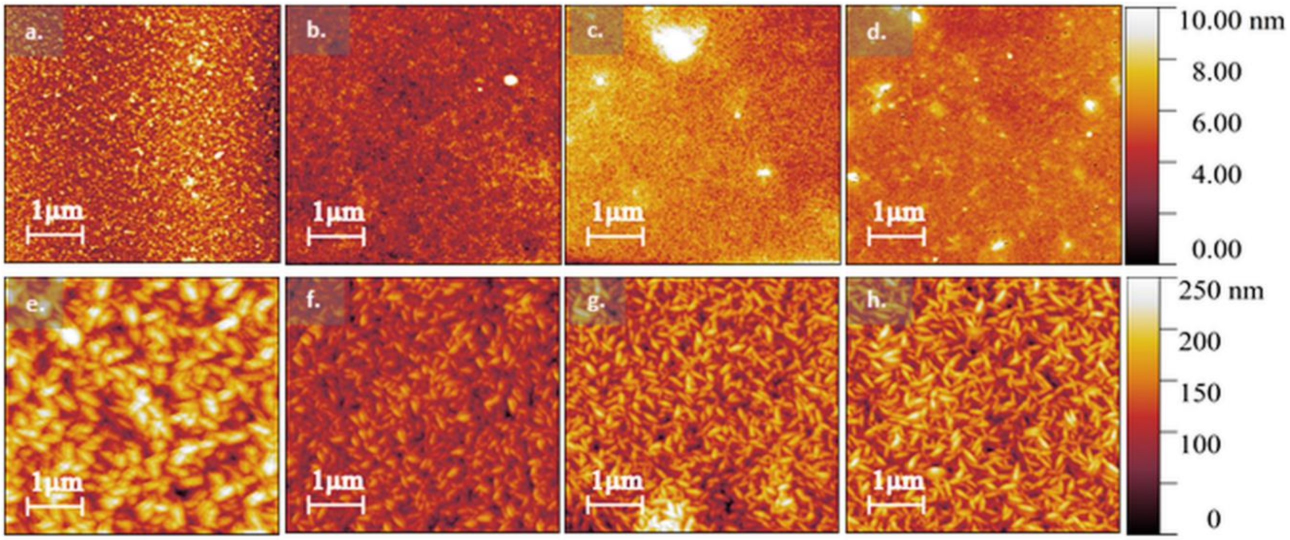

Figure S3. AFM images of the OSC a) P3HT, b) PCDTPT, c) PDPP4T, and d) DPPDTT and their corresponding heterojunctions with BiOI in the same order of the polymers is shown in e), f), g) and h).

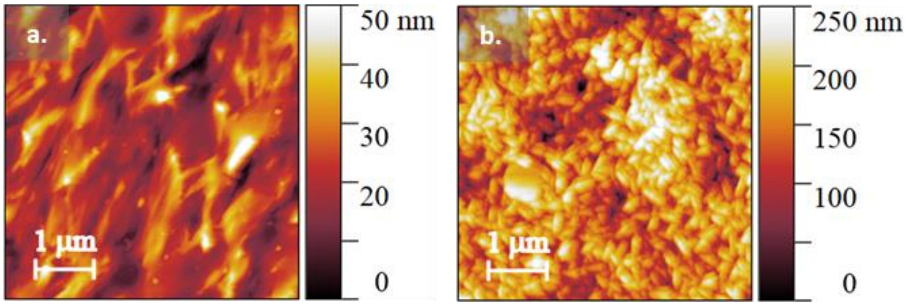

Figure S4. AFM images of the OSC a) TIPS- Pentacene and its corresponding heterojunction with BiOI in b).

UV-vis-NIR spectroscopy

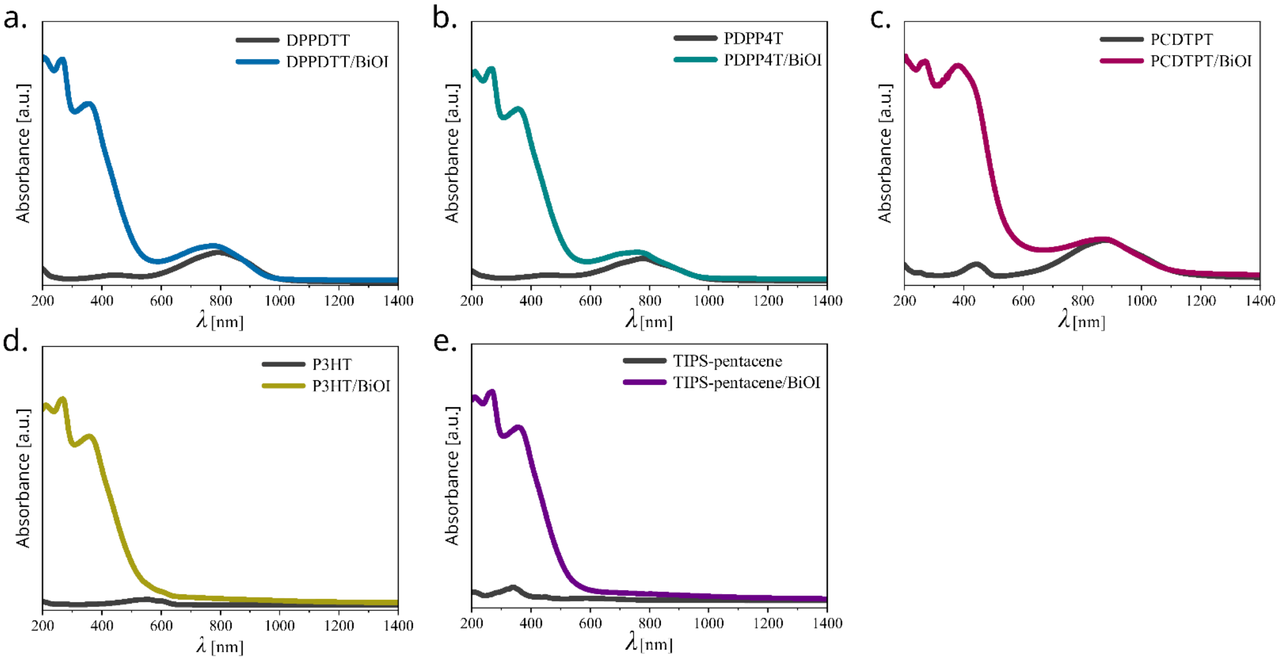

Figure S5. UV-vis-NIR absorption spectra of OSCs and OSC/BiOI heterojunctions showing the combined absorption of BiOI with the OSC a) DPPDTT, b) PDPP4T, c) PCDTPT, d) P3HT and d) TIPS-pentacene.

Device measurements

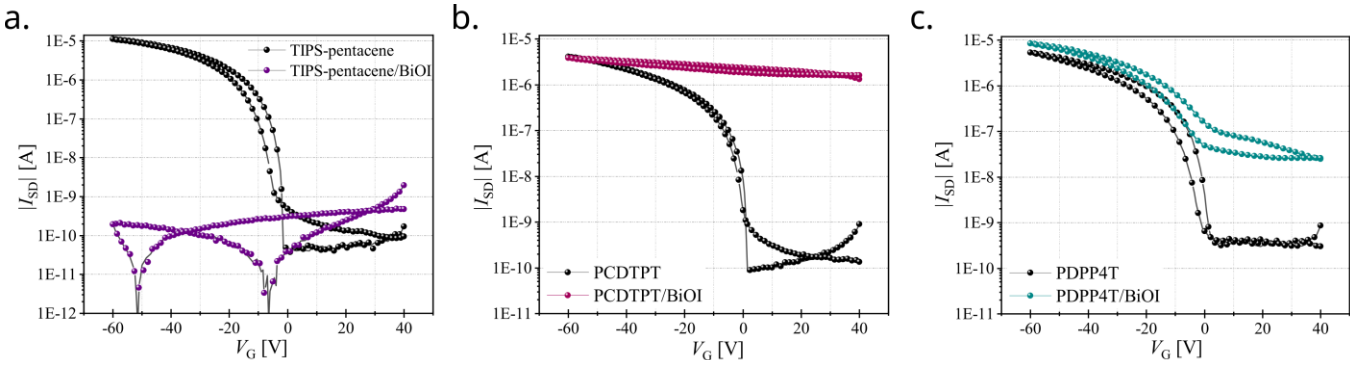

Figure S6. Transfer characteristics of pristine and heterojunction devices with a) TIPS-pentacene showing a collapse of device behavior, b) PCDTPT and c) PDPP4T.

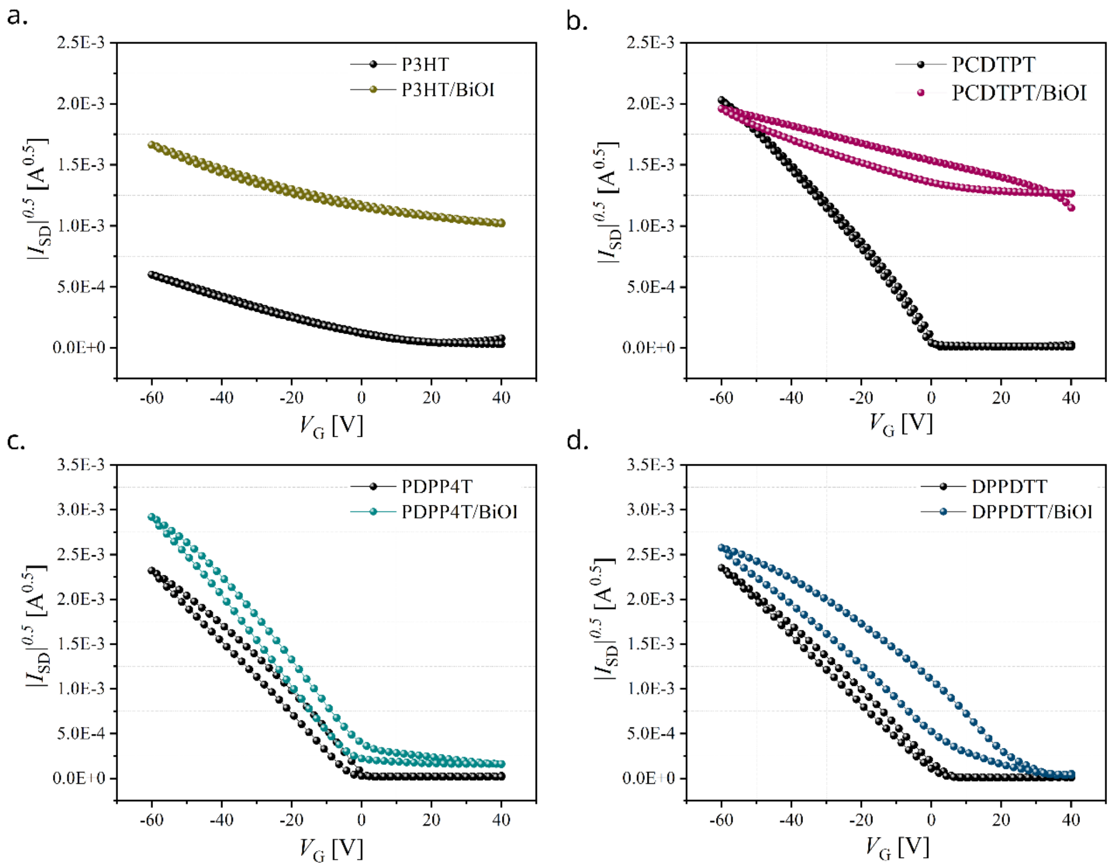

Figure S7. Square root plots of pristine OSCs and OSC / BiOI devices with a) P3HT, b) PCDTPT, c) PDPP4T and d) DPPDTT.

Table S1: The materials used with their respective supplier information and the HOMO- LUMO band positions as determined by UPS and UV-Vis-NIR spectroscopy.

**Material**	$\bar{M}_n$ [g·mol^{-1}]	$\bar{M}_w$ [g·mol^{-1}]	**PDI**	**Supplier**	**HOMO [eV]**	**Bandgap [eV]**	**LUMO [eV]**
**P3HT (97.3% RR)**	35 240	74 000	2.1	Ossila	–4.69	1.9	–2.79
**PCDTPT**	—	65 000	2.0	1-Material	–4.71	1.2	–3.51
**DPPDTT**	44 529	110 111	2.47	Ossila	–5.08	1.3	–3.78
**PDPP4T**	48 950	89 700	1.83	Ossila	–5.20	1.3	–3.90
**TIPS-pentacene**	—	639	—	Ossila	–5.14	1.5	–3.64

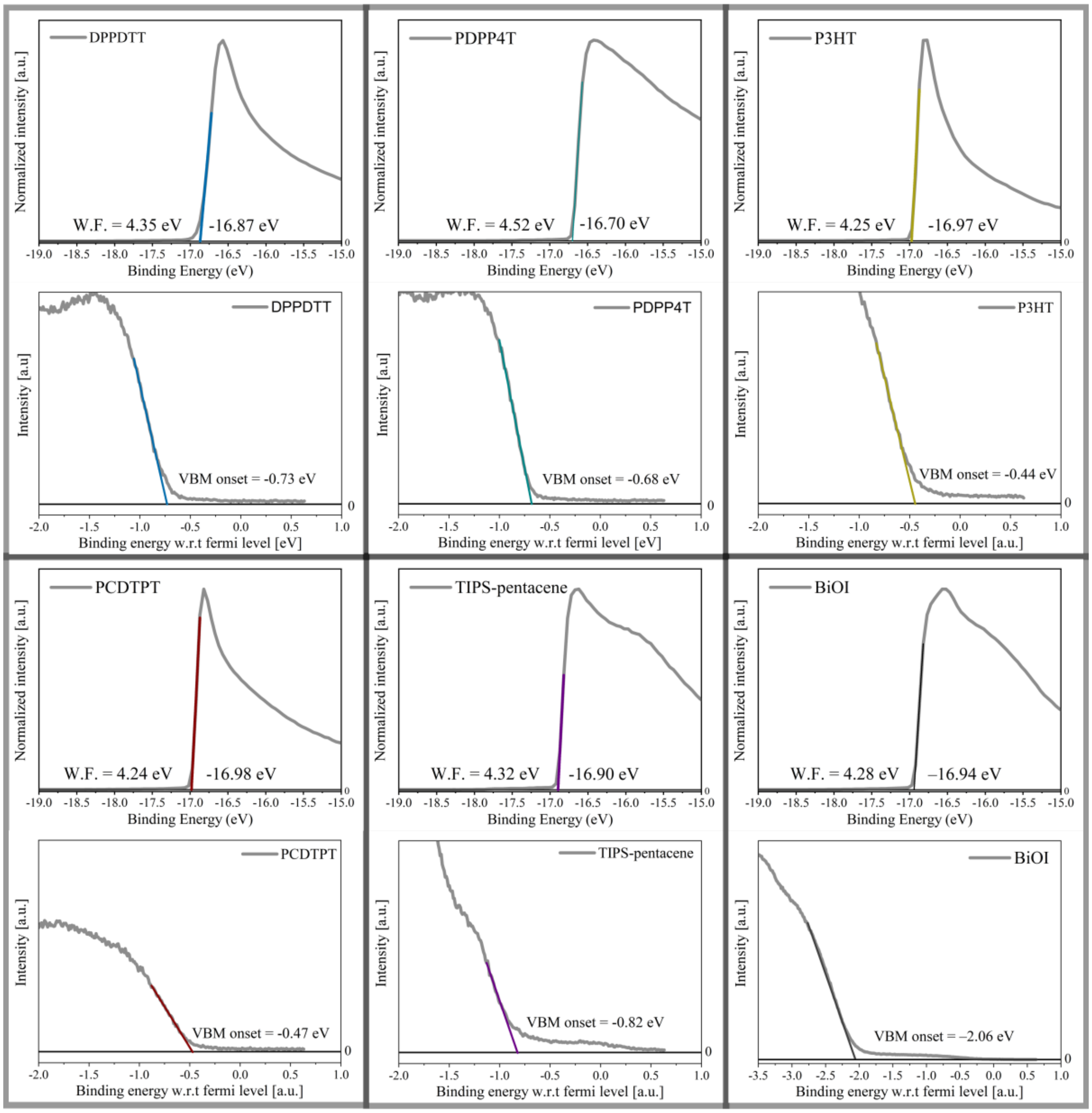

Figure S8. UPS spectra for OSC thin films used for the calculation of work function (W.F.) and valence band maximum (VBM) values.

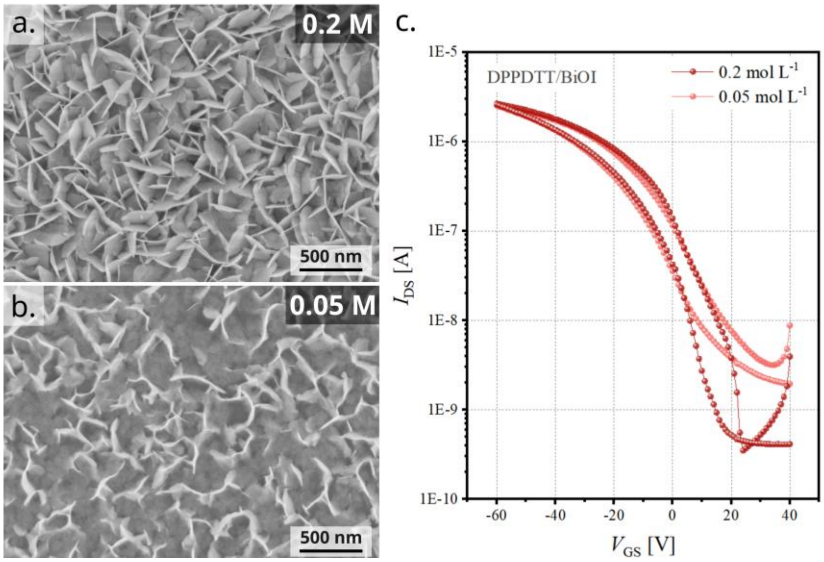

Figure S9. Top view SEM image of BiOI thin film coated from a) 0.2 mol L^{-1} and b) 0.05 mol L^{-1} BiI_3 precursor solution and c) transfer curves of the heterojunction FETs of the samples of coated using two different concentrations of BiI_3 solution.

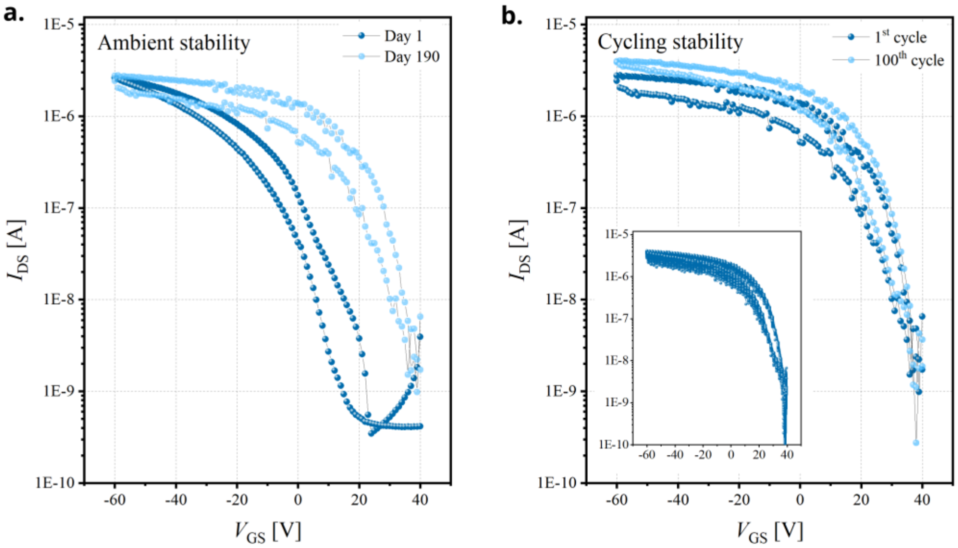

Figure S10. a) Ambient stability for over 6 months and b) cycling stability for 100 cycles (inset: all 100 cycles) of the DPDTT/BiOI heterojunction FET.
